# KIT promotes tumor stroma formation and counteracts tumor-suppressive TGFβ signaling in colorectal cancer

**DOI:** 10.1038/s41419-022-05078-z

**Published:** 2022-07-16

**Authors:** Emre Küçükköse, Niek A. Peters, Inge Ubink, Veere A. M. van Keulen, Roxanna Daghighian, André Verheem, Jamila Laoukili, Onno Kranenburg

**Affiliations:** Laboratory Translational Oncology, Division of Imaging and Cancer, University Medical Center Utrecht, 3584 CX Utrecht, The Netherlands

**Keywords:** Colorectal cancer, Cancer microenvironment

## Abstract

Expression profiling has identified four consensus molecular subtypes (CMS1-4) in colorectal cancer (CRC). The receptor tyrosine kinase KIT has been associated with the most aggressive subtype, CMS4. However, it is unclear whether, and how, KIT contributes to the aggressive features of CMS4 CRC. Here, we employed genome-editing technologies in patient-derived organoids (PDOs) to study KIT function in CRC in vitro and in vivo. CRISPR-Cas9-mediated deletion of the *KIT* gene caused a partial mesenchymal-to-epithelial phenotype switch and a strong reduction of intra-tumor stromal content. Vice versa, overexpression of *KIT* caused a partial epithelial-to-mesenchymal phenotype switch, a strong increase of intra-tumor stromal content, and high expression of TGFβ1. Surprisingly, the levels of phosphorylated SMAD2 were significantly lower in KIT-expressing versus KIT-deficient tumor cells. In vitro analyses showed that TGFβ signaling in PDOs limits their regenerative capacity. Overexpression of *KIT* prevented tumor-suppressive TGFβ signaling, while *KIT* deletion sensitized PDOs to TGFβ-mediated growth inhibition. Mechanistically, we found that KIT expression caused a strong reduction in the expression of SMAD2, a central mediator of canonical TGFβ signaling. We propose that KIT induces a pro-fibrotic tumor microenvironment by stimulating TGFβ expression, and protects the tumor cells from tumor-suppressive TGFβ signaling by inhibiting SMAD2 expression.

## Introduction

Colorectal cancer (CRC) remains a major cause of cancer-related mortality. Large scale gene expression profiling has recently identified four consensus molecular subtypes (CMS1-4) in CRC [1]. CMS1 mostly consists of tumors with a deficient mismatch repair (dMMR) system causing a hypermutated genome. CMS2, the ‘canonical subtype’ is characterized by activation of the Wnt pathway, and largely consists of chromosomally instable tumors. CMS3 is characterized by dysregulation of metabolic pathways. Finally, CMS4 tumors are characterized by high expression of genes reflecting epithelial-to-mesenchymal transition (EMT), transforming growth factor β (TGFβ) signaling, and matrix remodeling. CMS4 tumors also have a high stromal cell content. Furthermore, CMS4 is associated with poor prognosis and a poor response to systemic therapy [1–5]. Clearly, the development of effective CMS4-targeting therapies is an unmet clinical need.

Recently, we found that the genes encoding platelet-derived growth factor receptor (PDGFR) type B and the stem-cell factor (SCF) receptor (KIT) are highly expressed in CMS4 colon tumors [6]. The expression of PDGFRs in tumors correlates with an unfavorable prognosis in CRC as well as various other types of cancer [7–10]. Inhibition of PDGFR signaling limits CRC invasion and the formation of distant metastases [11–13]. Much less is known about the role of KIT in CRC. KIT is structurally closely related to PDGFRs and its expression is mostly restricted to primitive stem-like cell types [14]. KIT can promote cell growth, survival, migration, differentiation, and secretion in different biological contexts [14–16]. Amplification and activating point mutations in *KIT* are well-documented in gastrointestinal stromal tumors (GISTs) and melanoma, but occur very infrequently in CRC [14, 16]. However, we and others have previously shown that signaling by (wild-type) *KIT* maintains stem-like cancer cells in CRC and is required for colony- and tumor-forming potential [17, 18].

How *KIT* expression promotes aggressive behavior of CRC cells and how its expression is correlated to the distinguishing features of CMS4 CRC, remains incompletely understood. Here, we studied the tumor cell-intrinsic role of KIT in three-dimensional patient-derived tumor organoids (PDOs) by generating PDO variants in which the *KIT* gene was either deleted (by CRISPR-Cas9-mediated gene editing), or overexpressed (by using a lentiviral expression construct). The modified PDOs were then used in in vitro and in vivo assays to study how *KIT* regulates various aspects of CMS4 CRC, including tumor stroma formation, TGFβ signaling, and EMT.

## Materials and methods

### Human tissue samples

Tissue sample from CRC patient (PDO1) was collected during surgery within the Biobanking protocol HUB-Cancer TCBIO #12-093, which was approved by the medical ethical committee of the University Medical Center Utrecht (UMCU). Written informed consent from the donor was obtained prior to the acquisition of the specimen for research use in the present study.

### In vitro organoid culture

Patient-Derived Organoid (PDO) #1 was generated in this study as described previously. PDO2 was obtained from Wetering et al. [19] (original nomenclature is p26T). The Tumor Progression Organoid models (TPO3 and TPO4) were obtained from Drost et al. [20]. Culturing organoids was performed by embedding in ice-cold Matrigel® (Corning, Corning, NY, USA), mixed with a CRC culture medium (Table S1) in a 3:1 ratio. For passaging, the tumor organoids were dissociated with TrypLE Express (Gibco, Breda, The Netherlands, #12604021) for 5–10 min at 37 °C and re-plated in a pre-warmed six-well plate. Rho-associated kinase (ROCK) inhibitor Y-27632 (Tocris, Abingdon, UK, #1254, 10 μM) was added to culture medium upon plating for two days.

### Genomic engineering: CRISPR-Cas9 mediated knock out and lentiviral overexpression

For overexpressing KIT in PDO2, TPO3, and TPO4, cDNA of *KIT* was derived from PDO1, cloned into pEGFP-N1 (Addgene #6085-1), and subsequently inserted into the lentiviral construct pWPT (Addgene #12255) using in-house primers (Table S2). For CRISPR-Cas9 mediated knock-out of *KIT* in PDO1, a single-guide RNA (Table S2) targeting exon 8 was ligated into LentiCRISPRv2 (Addgene #52961). Lentiviral production of above constructs was performed using a calcium phosphate transfection protocol in human embryonic kidney 293T cells using the transfer plasmid (15 µg), pMD2.G (#12259, 7.5 µg) and psPAX2 (#12260, 7.5 µg). The following day, medium was replaced by advanced DMEM/F12 medium (Invitrogen) supplemented with HEPES buffer (Lonza, 10 mM), penicillin/streptomycin (Gibco, 50 U/ml), and GlutaMAX (Gibco, 2 mM). The next day, 50,000 single cells of organoids were resuspended in the virus medium (which was filtered through a 0.45 μm polyethersulfone filter), supplemented with Polybrene (Sigma-Aldrich, 8 µg/ml), N-acetylcysteine (Sigma-Aldrich, 1.25 mM) and ROCK-inhibitor Y-27632 (Sigma-Aldrich, 10 μM), and incubated overnight 37 °C, 5% (vol/vol) CO_2_ on non-adherent plates (ultra-low attachment surface, Sigma-Aldrich). After 24 h incubation, cells were washed twice in PBS (Sigma-Aldrich) and cultured as described above. The PDOs were FACS-sorted based on KIT expression (Table S3) at least two passages after transduction using a Fluorescence Activated Cell Sorting (FACS) Aria II (BD Biosciences) machine.

### Regenerative capacity assay

The tumor organoids were dissociated into single-cells by incubating in TryPLE Express for 5 min at 37 °C. Single cells were counted and cell suspensions were prepared with 20,000 cells/mL in CRC culture medium. A volume of 100 µl Matrigel was added to a well of six-well plate and subsequently, 50 µl of cell suspensions (i.e., total 1000 cells) was added to the matrigel droplet and mixed by pipetting. The mixture was spread through the surface of the well by circular moves. CRC culture medium was added to the wells after the cell-matrigel suspensions was solidified at 37 °C for 15 min. Medium was refreshed twice a week. Each condition was seeded in minimal triplicates per assay. Clones were counted two weeks after the cell seeding and quantified relative to the control.

### Statistical analyzes

Statistical analysis and graphs were made using R software version 4.0.2. The analyses were performed using unpaired t-tests and *p*-values of <0.05 were considered significant. Values are presented as mean ± standard error of the mean. For all figures, the statistical test are justified as appropriate and the data meet the assumptions of the tests. Estimates of variation are included for each group of data and are reported in the corresponding figure legends. The variance is similar between groups that are being statistically compared.

## Results

### KIT co-expressed genes identify a subgroup of mesenchymal-like CRC

**Fig. 1:**
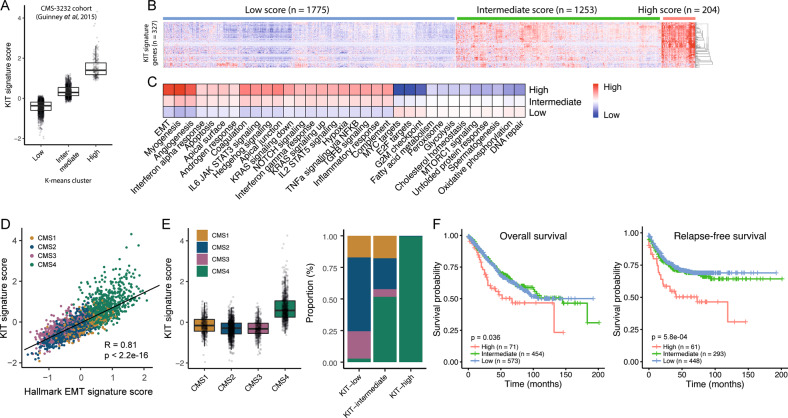
KIT co-expressed genes identify a subgroup of metastasis-prone tumors and are strongly correlated with CMS4. **A** Boxplot showing the “KIT-co expressed signature” score for KIT-low, KIT-intermediate, and KIT-high groups in the large transcriptome cohort (*n* patients = 3232) [1]. The groups are clustered by using the k-means algorithm (*n* = 3). **B** Heat map showing the expression levels of 327 genes in the “KIT-co expressed signature” for the three groups. **C** Gene set enrichment analyses of hallmark signatures for KIT-low, KIT-intermediate, KIT-high annotated tumors. **D** Scatter plot showing the correlation of “KIT-co expressed signature” expression values in relation to the hallmark Epithelial-Mesenchymal-Transition signature. **E** Boxplot showing “KIT-co expressed signature” score per CMS-type and stacked bar plot showing the CMS distribution in KIT-annotated subgroups. **F** Kaplan–Meier curves showing overall- (left) and relapse-free (right) survival in tumor subgroups defined by the “KIT-co expressed signature” in the CMS3232 cohort [1]. A two-sided log-rank test was applied to assess the significance of the survival differences between the two groups.

To gain insight into the clinical impact of *KIT* expression in CRC, we applied a previously described “*KIT-co-expression signature*”, containing the top-327 genes that are most significantly co-expressed with *KIT* in CRC [17]. This gene set was used to cluster a large CRC cohort (*n* patients = 3232; [1]) into three *KIT*-expression subgroups (low, intermediate, and high) by using the k-means algorithm (Fig. 1A, B). Gene set analysis using the signatures in the Molecular Signatures Database (MSigDB) revealed a highly significant positive correlation of the KIT-signature with the hallmark EMT signature (*r* = 0.81 and *p* < 2.2e−16) (Fig. 1C, D). Moreover, 99.5% of the tumors in the KIT-high subgroup were classified as CMS4, further confirming the high expression of *KIT* in mesenchymal tumors (Fig. 1E). Indeed, patients in the KIT-high subgroup were significantly more prone to develop recurrence than patients in the KIT-intermediate and KIT-low groups (*p* = 5.8e−04) (Fig. 1F).

### KIT promotes the regenerative capacity of patient-derived tumor organoids

**Fig. 2:**
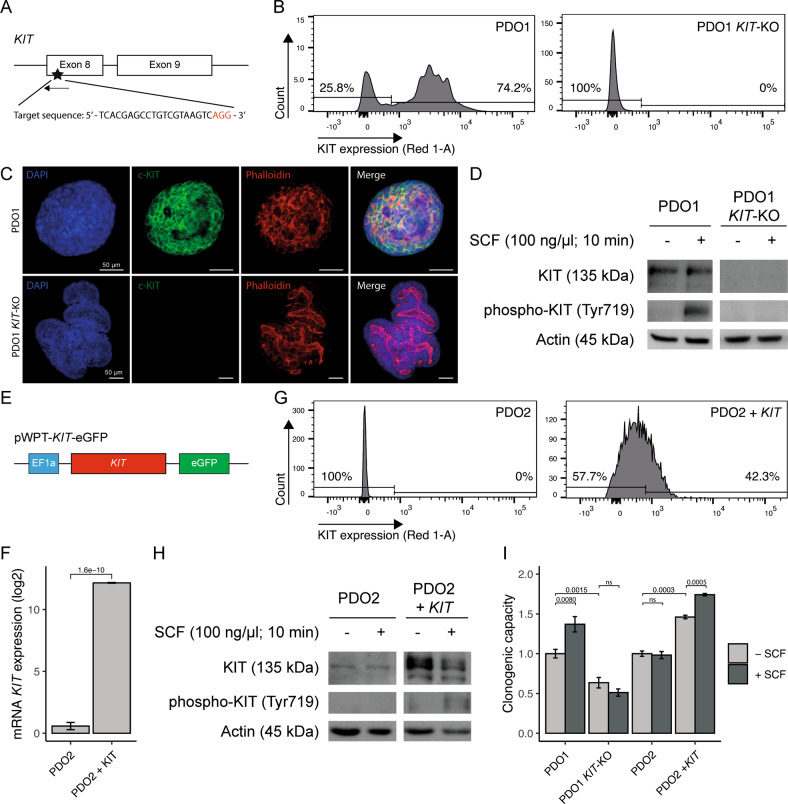
Genomic engineering of *KIT* in patient-derived tumor organoids increases regenerative capacity. **A** Schematic overview of CRISPR-Cas9 mediated *KIT* gene knockout in PDO1. Designed single-guide RNA targets *KIT* exon 8. **B** Flow-cytometry analyses showing deletion of cell-surface KIT expression in PDO1^KIT-KO^. **C** Immunofluorescence imaging demonstrating DAPI, c-KIT, and Phalloidin staining in PDO1^CONTROL^ and PDO1^KIT-KO^, scale bar is 50 µm. **D** Western blot analysis of KIT and phosphorylated KIT upon stimulation with stem-cell factor (SCF, 100 ng/µl) for 10 min in PDO1^CONTROL^ and PDO1^KIT-KO^. **E** Schematic overview of lentiviral vector to overexpress KIT, which is driven by the human EF1a promotor in PDO2. **F** mRNA levels of KIT expression in PDO2^CONTROL^ and generated PDO2^KIT^ variant. **G** Flow-cytometry analyses showing presence of cell-surface KIT expression in PDO2^KIT^. **H** Western blot analysis of KIT and phosphorylated KIT upon stimulation with stem-cell factor (SCF, 100 ng/µl) for 10 min in PDO2^CONTROL^ and PDO2^KIT^. **I** Regenerative capacity of PDOs with KIT variants was assessed by counting the number of regenerated organoids. At least two independent experiments with three technical replicates. An unpaired t-test was applied to assess the significance between the groups.

To start exploring the function of *KIT* in CRC, we applied CRISPR-Cas9-mediated genome editing of *KIT* exon 8 in patient-derived organoid 1 (PDO1) in order to knock-out the gene (Fig. 2A). KIT-negative cells were isolated using fluorescence-activated cell sorting (FACS) (Fig. 2B). Immunofluorescence and Western-blot analysis confirmed loss of *KIT* expression in PDO1^KIT-KO^ (Fig. 2C, D). Stimulation of PDOs with the KIT ligand stem cell factor (SCF) resulted in KIT phosphorylation on Y719 in wild-type PDO1^CONTROL^, but not in PDO1^KIT-KO^ (Fig. 2D).

Next, we chose a PDO lacking endogenous KIT expression (PDO2) and transduced it with a lentiviral vector in which *KIT* expression is driven by the human promotor EF1a (Fig. 2E). KIT-expressing cells were purified using FACS sorting and expressed high levels of *KIT* mRNA (Fig. 2F, G). Stimulation with SCF resulted in *KIT* phosphorylation on Tyr719 in PDO2^KIT^, but not in control PDO2^CONTROL^ (Fig. 2H).

Previously we demonstrated that SCF stimulation increased the regenerative capacity of patient-derived three-dimensional ‘spheroid’ cultures, whereas KIT inhibitors reduced their regenerative capacity [17]. Therefore, we first tested how deletion or overexpression of *KIT* would influence regenerative capacity of the newly generated PDOs. To test this PDO1^CONTROL^, PDO^KIT-KO^, PDO2^CONTROL^, and PDO2^KIT^ were seeded as single cells in the absence or presence of SCF, and their regenerative capacity (i.e., the number of regenerated organoids) was scored two weeks later. These analyses revealed that *KIT* deletion reduced the regenerative capacity of PDO1^KIT-KO^ and rendered cells insensitive to SCF (Fig. 2I). Vice versa, overexpression of *KIT* in PDO2^KIT^ caused an increased regenerative capacity, which was further stimulated by SCF addition (Fig. 2I).

### KIT promotes tumor stroma formation

**Fig. 3:**
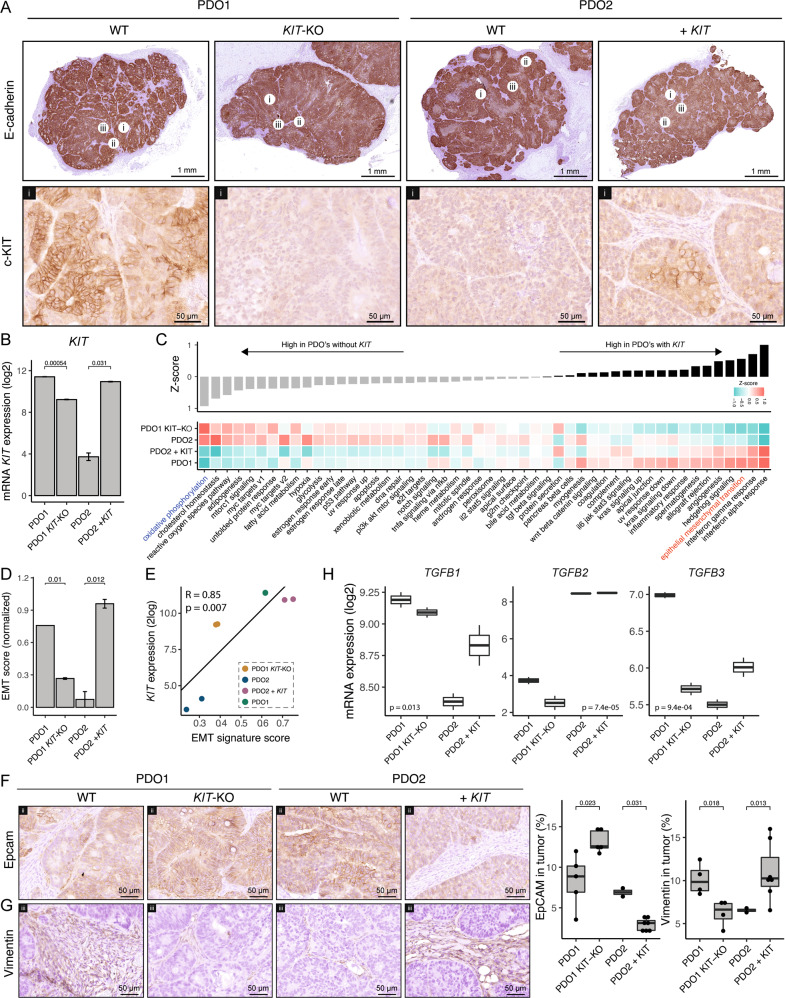
KIT induces stroma formation in PDO-initiated subcutaneous tumors in NSG mice. **A** Histological overview of PDO1^CONTROL^, PDO1^KIT-KO^, PDO2^CONTROL^, and PDO2^KIT^ initiated subcutaneous (s.c.) tumors in NSG mice (*n* = 2 per group). Expression of epithelial-junction marker E-cadherin and cell-surface KIT are shown. Circles with numbers indicate the region of zoom-in images. Scale bar for E-cadherin is 1 mm and for KIT 50 µm. **B** mRNA level of KIT in s.c. tumors. **C** Gene set enrichment analyses of hallmark signatures for the s.c. tumors, ranked based on Z-score. **D** Barplot showing the hallmark EMT signature score for each s.c. tumor. **E** Scatter plot showing positive correlation with *KIT* expression and EMT score for s.c. tumors. **F** Histological images of a epithelial marker EpCAM and (**G**) stroma-marker Vimentin in s.c. tumors. Box plots show the quantified expression of EpCAM and Vimentin in a s.c. tumors (**H**) mRNA levels of TGFB1-3 ligands in s.c. tumors. An unpaired t-test was applied to assess the significance between the groups.

Next, we explored the effect of *KIT* deletion or overexpression in vivo. Subcutaneous injection of PDO1^CONTROL^, PDO1^KIT-KO^, PDO2^CONTROL^, and PDO2^KIT^ into NSG mice resulted in tumor formation in all groups. KIT expression was clearly detected by immunohistochemistry (IHC) in PDO1^CONTROL^ tumors and in PDO2^KIT^ tumors. By contrast, tumors formed by PDO1^KIT-KO^ and PDO2^CONTROL^ lacked expression of KIT (Fig. 3A). Expression of the epithelial junction marker E-cadherin was not significantly different between KIT expression subgroups (Fig. 3A). To gain insight into how KIT influences tumor biology, RNA was isolated from two tumors of all PDO variants and sent for RNA sequencing. Differential gene expression analysis confirmed significant differences in *KIT* mRNA levels between the models (Fig. 3B). The residual expression of *KIT* mRNA in PDO1^KIT-KO^ is due to the fact that exon 1-7 are not targeted by the knockout strategy, and may result in expression of a truncated mRNA that can be detected by sequencing. Indeed, differential exon expression analysis confirmed decreased exon usage after exon 8, which is targeted by the knockout strategy (Fig. S1). Gene set enrichment analyses using 50 cancer hallmark signatures revealed a surprisingly concordant effect of altering *KIT* expression in these two completely distinct PDO models (Fig. 3C). Signatures reflecting inflammatory processes (interferon alpha, interferon gamma, inflammatory response) and stroma activation (angiogenesis, hedgehog signaling) were upregulated in KIT-expressing tumors (PDO1^CONTROL^ and PDO2^KIT^) compared to tumors lacking KIT expression (PDO1^KIT-KO^, PDO2^CONTROL^) (Fig. 3C). Interestingly, the Hallmark Epithelial-Mesenchymal-Transition signature was also significantly higher in KIT-expressing tumors (Fig. 3C–E). We recently showed that partial EMT with the maintenance of E-cadherin expression yields epithelial cells in a quasi-mesenchymal state, which display reduced expression of EpCAM [21]. Therefore, we analyzed expression of EpCAM in tumors expressing KIT *versus* those that do not. EpCAM expression was significantly reduced in tumors expressing KIT (PDO1^CONTROL^ and PDO2^KIT^) compared to those without KIT (PDO1^KIT-KO^ and PDO2^CONTROL^), while E-cadherin levels remained constant, indicative of a partial EMT (Fig. 3F).

Mesenchymal gene expression in CRC has been largely attributed to infiltrating stromal fibroblasts [22, 23]. Therefore, we performed IHC staining for the fibroblast marker vimentin on tumor tissue sections. This revealed a significantly higher percentage of vimentin-positive stroma in KIT-expressing tumors *versus* those lacking KIT expression (Fig. 3G). One of the most potent pro-fibrotic cytokines is TGFβ. We found that expression of TGFβ1 and TGFβ3 genes significantly increased following KIT overexpression, while expression of both genes was reduced following KIT deletion (Fig. 3H). IHC confirmed the increase of TGFb1 protein level in KIT-positive tumors (Fig. S2). These results directly link KIT expression to high stromal content.

Notably, we observed that tumors lacking KIT expression displayed significantly higher expression of the signature reflecting “oxidative phosphorylation” (Fig. 3C), which is known to fuel the anabolic needs of proliferating epithelial-like tumors [24]. Indeed, expression of oxidative phosphorylation and EMT signatures were inversely correlated (*r* = −0.98 and *p* = 1.4e−05) both in the KIT-positive PDO models (Fig. S3) and in the large cohort of primary CRC (*r* = −0.37 and *p* < 2.2e−16).

**Fig. 4:**
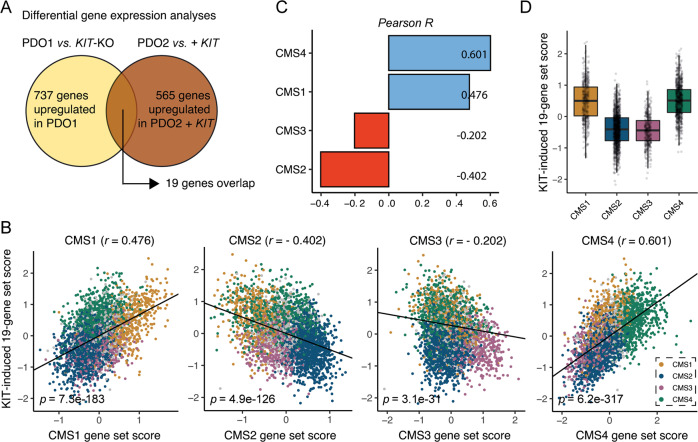
KIT causes a shift from epithelial (CMS2) to mesenchymal (CMS4) phenotype. **A** Differential gene expression analysis identified 19 overlapping genes that were significantly higher expressed in PDOs expressing KIT compared to those without KIT expression. **B** Scatter plots showing the correlation of the identified KIT-dependency gene signature with CMS1-4-identifying genes in the CMS-3232 CRC cohort [1]. Bar plot showing Pearson *R* values of (**C**). **D** Score for the KIT-dependency gene signature per CMS-annotated tumor in the CMS-3232 cohort.

Taken together, these data show that *KIT* expression causes a phenotype change resembling a shift from CMS2 (epithelial-like) to CMS4 (partial EMT; high stroma). To test this directly, we first identified a core set of 19 genes that were both significantly induced by KIT overexpression *and* significantly reduced following KIT deletion (Fig. 4A and Table S4). Expression of this KIT-dependency gene signature correlated extremely well with the “KIT-co-expressed signature” [17] (Fig. S4) and CMS4-identifying genes in the golden standard random forest CMS classifier (*r* = 0.601), less so with CMS1-identifying genes (*r* = 0.476) and negatively with CMS3- and CMS2-identfying genes (*r* = *−*0.202 and *r* = −0.402 respectively) (Fig. 4B–D).

### KIT counteracts tumor-suppressive TGFβ-signaling

**Fig. 5:**
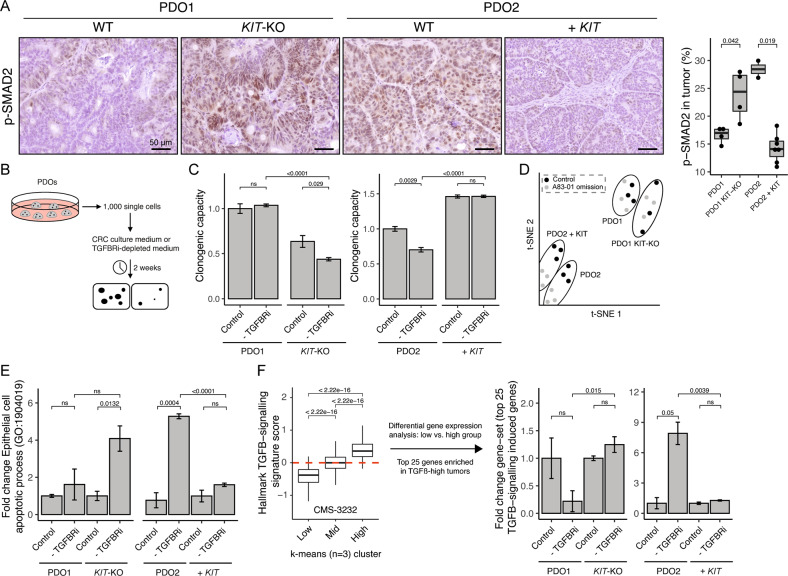
KIT counteracts tumor-suppressive TGFβ-signaling. **A** Histological images of p-SMAD2 in s.c. tumors and corresponding quantification. **B** Schematic overview of the regenerative capacity assay, in which 1000 single cells are seeded in CRC culture medium or A83-01 depleted medium. The number of organoids are counted after two weeks. **C** Quantification of the regenerative capacity assay as described in (**B**), at least two different experiments with three technical replicates in each. **D** t-SNE projection of RNA-sequencing data of in vitro PDOs, grown in CRC culture medium (control) or TGFBRi-depleted (A83-01 omission) medium. **E** Bar plot showing fold change of “Epithelial cell apoptotic process (GO: 1904019)”. **F** A “TGFB-response” gene set is generated by using the k-means algorithm and clustering the CMS-3232 cohort into three groups, defined by low-, intermediate- and high expression for the hallmark TGFB-signaling signature. The score for the top 25 genes upregulated in the TGFB-High group is assessed in the RNA-sequencing data of the regenerative capacity assay in (**C**). An unpaired t-test was applied to assess the significance between the groups.

One of the most characteristic features of CMS4 CRC is a high level of TGFβ signaling in the tumor stroma (Fig. 1C) [1]. However, the role of tumor-intrinsic TGFβ-signaling in CRC cells is incompletely understood. This is important because TGFβ signaling in epithelial (tumor) cells can have either a tumor-suppressive or a tumor- and metastasis- promoting (invasion/EMT) effect [25]. As TGFβ expression is induced following KIT overexpression and lost following KIT deletion (Fig. 3H), we tested how *KIT* expression influences canonical TGFβ signaling in tumor cells. To this end, we analyzed SMAD2 phosphorylation levels in tumors with or without KIT expression by IHC. Strikingly, despite the high expression of TGFβ and the high stromal content in KIT-expressing tumors, we observed a significantly lower level of SMAD2 phosphorylation in the neoplastic cells of such tumors, when compared to control tumors without *KIT* (Fig. 5A).

To study how KIT expression regulates tumor cell-intrinsic TGFβ signaling, we used the various PDO cultures. One of the components in PDO growth medium is the TGFβ receptor inhibitor A83-01, to counteract potential growth-suppressive effects of TGFβ signaling during PDO establishment and expansion. Indeed, we found that omitting A83-01 from the culture medium (allowing TGFβ signaling to occur) had a small but significant inhibitory effect on the regenerative capacity of PDO2^CONTROL^ (Fig. 5B, C). However, expression of KIT in PDO2^KIT^ not only increased their basal regenerative capacity, but also rendered them insensitive to TGFβ-mediated growth inhibition (A83-01 omission). Likewise, PDO1^CONTROL^ (expressing KIT) were insensitive to omission of A83-01 from the culture medium, but knockout of *KIT* rendered PDO1^KIT-KO^ sensitive to TGFβ-mediated growth inhibition (Fig. 5C and Fig. S5). Similar to KIT knockout, treatment with the KIT inhibitors Dasatinib or Imatinib significantly reduced the regenerative capacity of PDO1 in the presence of SCF. Secondly, we found that the regenerative capacity of PDO1 was even further reduced if A83-01 was omitted from the culture medium, allowing TGFβ signaling to occur. Thus, both KIT inhibitors, similar to KIT knockout, sensitized PDO1 to TGFβ-mediated growth inhibition (Fig. S6).

Next, we performed RNA sequencing of all PDO variants grown in the presence or absence of A83-01. T-SNE algorithm separated PDO models but not culture condition (control and A83-01 omission) (Fig. 5D). Analysis of the expression data revealed that expression of the gene ontology signature “epithelial cell apoptotic process” (GO: 1904019) increased significantly following A83-01-depletion (allowing TGFβ signaling) in PDOs lacking KIT expression, but not in KIT-expressing PDOs (Fig. 5E). The changes in gene expression following A83-01 omission in PDOs without KIT presumably reflect activation of TGFβ signaling. To further substantiate this, we first clustered CRC tumors in the CMS3232 cohort [1] into three subgroups based on the TGFβ-Hallmark signature (low, intermediate, and high) using the k-means algorithm and subsequently performed differential gene expression analysis between TGFβ-low *vs*. TGFβ-high tumors (Fig. 5F). The top 25 genes that were most significantly upregulated in TGFβ-high clinical samples were significantly induced following A83-01 omission in PDOs lacking KIT expression, but not in KIT-expressing PDOs (Fig. 5F and Table S5). Thus, a gene set derived from clinical samples distinguishing TGFβ-high from TGFβ-low tumors, was activated by A83-01 omission from the PDO culture medium, and suppressed by KIT expression.

**Fig. 6:**
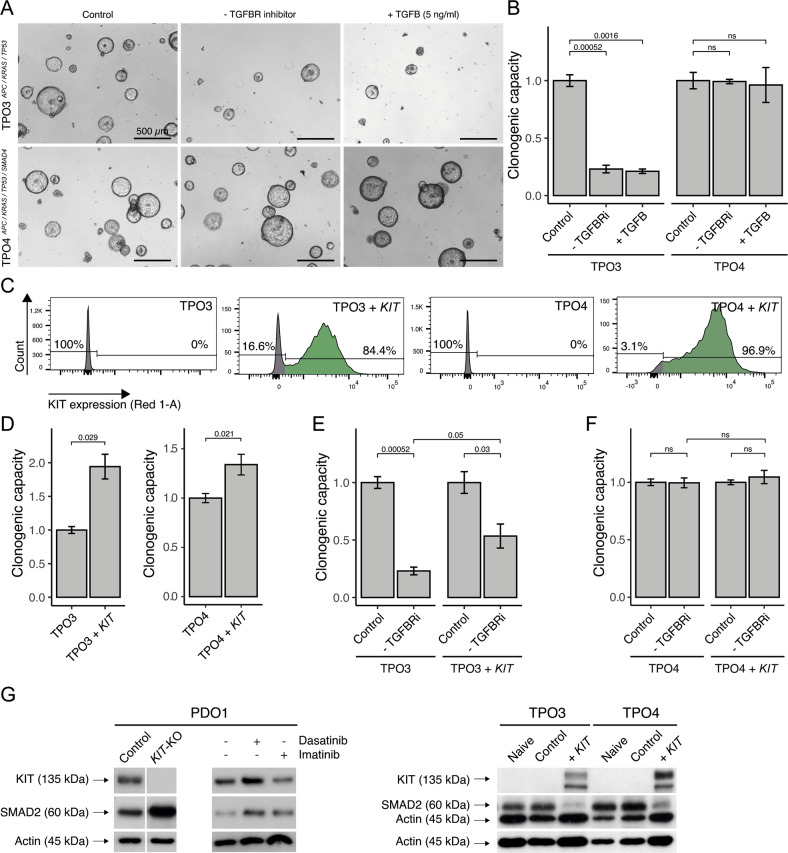
KIT expression partially substitutes for SMAD4 mutation in tumor progression organoids. **A** Brightfield images of TPO3 and TPO4 organoids in normal CRC culture medium, A83-01 depleted medium, and/or TGFB-stimulated medium (5 ng/ml). Scale bar is 500 µm. **B** Regenerative capacity assay of (**A**). **C** Flow-cytometry analysis demonstrating presence of KIT in TPO3^KIT^ and TPO4^KIT^, generated with lentiviral overexpressing vector. **D** KIT increases the regenerative capacity in TPO3^KIT^ and TPO4^KIT^, at least two experiment with three technical replicates in each. **E** Regenerative capacity assay for TPO3^CONTROL^, TPO3^KIT^, **F** TPO4^CONTROL^, and TPO4^KIT^ in normal CRC culture medium and A83-01 depleted medium, at least two experiment with three technical replicates in each. **G** Western blot demonstrating reduced total SMAD2 protein in organoids with KIT expression. Dasatinib 100 nM and Imatinib 5 µM. An unpaired t-test was applied to assess the significance between the groups.

The tumor-suppressive effects of TGFβ signaling can be modeled in a series of ‘tumor-progression organoids (TPO)’ in which PDOs from healthy intestine are transformed in a stepwise fashion by CRISPR-CAS9-mediated introduction of mutations in *APC*, *KRAS*, *TP53,* and *SMAD4* [20]. In this TPO series, *SMAD4* deletion (blocking TGFβ signaling) increases tumorigenic and metastatic capacity [26]. We found that omission of A83-01 from the medium of triple mutant organoids (TPO3: *APC*, *KRAS*, *TP53*) resulted in a near-complete cessation of regenerative capacity, while quadruple mutant organoids (TPO4: *APC*, *KRAS*, *TP53*, and *SMAD4*) were not affected (Fig. 6A, B). Furthermore, two distinct TGFBR inhibitors (A83-01 and Galunisertib [27]) stimulated the regenerative capacity of PDO2 and TPO3 (wildtype TGFb pathway and lacking KIT expression) to a similar extent, while neither inhibitor affected the regenerative capacity of PDO1 (expressing KIT) or TPO4 (deficient TGFβ patwhay). (Fig. S7). Exogenous addition of TGFβ did not further aggravate the growth-suppressing effect of A83-01 omission (Fig. 6A, B). Next, we transduced TPO3 and TPO4 with the lentiviral KIT expression construct (Fig. 2E), yielding TPO3^KIT^ and TPO4^KIT^ organoids. Flow cytometry analysis demonstrated cell surface expression of KIT in both models (Fig. 6C). TPO3^KIT^ and TPO4^KIT^ organoids displayed a significant ~two-fold increased regenerative capacity in the presence of A83-01 (Fig. 6D). Importantly, KIT expression also—partially—rescued the growth-suppressive effect caused by TGFβ signaling (A83-01 omission; Fig. 6E). Moreover, the regenerative capacity of SMAD4-mutated TPO4^KIT^ was unaffected by omission of A83-01 (Fig. 6F).

Western blot analysis of organoid lysates showed that KIT expression in TPO3^KIT^ and TPO4^KIT^ strongly reduced SMAD2 expression levels, while KIT knockout in PDO1, or treatment of PDO1 with the KIT inhibitors Dasatinib or Imatinib caused a marked increase in SMAD2 levels (Fig. 6G). This provides insight into how KIT protects tumor cells against the tumor suppressive effects of TGFβ.

### KIT expression promotes the formation of stroma-rich tumors in the mouse caecum

**Fig. 7:**
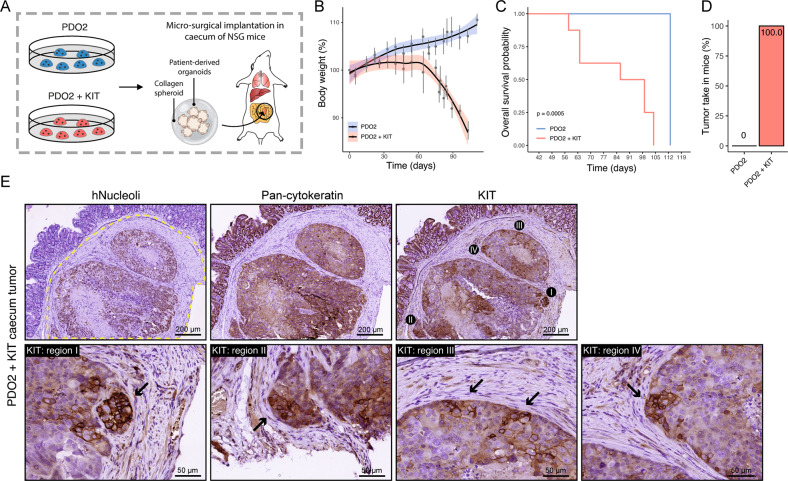
KIT expression promotes the formation of stroma-rich tumors in the mouse caecum. **A** Schematic overview of orthotopic implantation of PDO2^CONTROL^ and PDO2^KIT^ in the submucosa of NSG caecum. **B** Body weight during the experiment, day 0 is start of the experiment. **C** Kaplan–Meier curve demonstrating overall survival in PDO2^CONTROL^ and PDO2^KIT^ implanted mice. **D** Tumor take in mice, i.e., successful engraftment of implantation and formed primary tumors. **E** Histological images of human Nucleoli, pan-Cytokeratin, and KIT in primary tumors derived from mice, which were implanted with PDO2^KIT^. Scale bars are 200 and 50 µm.

Finally, we assessed whether KIT expression would promote the tumorigenic capacity of PDO2. To this end, we performed orthotopic implantation of PDO2^CONTROL^ or PDO2^KIT^ in the submucosa of the caecum of NSG mice (*n* = 7 and 8 per group, respectively) (Fig. 7A) [28]. Weight loss over time (from 60 days onwards) was observed in mice transplanted with PDO2^KIT^ organoids, but not in those transplanted with PDO2^CONTROL^ (Fig. 7B). The median survival (until the humane endpoint) was 93 days in mice transplanted with PDO2^KIT^ organoids whereas none of the mice transplanted with PDO2^CONTROL^ reached the humane endpoint (Fig. 7C). The latter group was sacrificed 113 days post-surgery for histological analysis. Tumor take in mice (*n* = 8) with PDO2^KIT^ implantation was 100%, whereas none of the mice (*n* = 7) transplanted with PDO2^CONTROL^ had primary tumors at the site of implantation (Fig. 7D). Immunohistochemistry for human Nucleoli, pan-cytokeratin and KIT confirmed the presence of KIT-expressing invasive stroma-rich carcinomas initiated by PDO2^KIT^ (Fig. 7E). Interestingly, the highest expression of KIT was found in tumor areas directly adjacent to the tumor stroma (Fig. 7E).

## Discussion

In this study, we show that *KIT* expression in CRC induces CMS4-like features, including a high tumor stroma content, high expression of TGFβ, accompanied by partial EMT and higher regenerative capacity of the tumor cells. Interestingly, KIT also provides protection against tumor-suppressive TGFβ signaling, possibly by reducing the expression of SMAD2. A high stromal content in (CMS4) CRC correlates with aggressive behavior, poor survival, and a poor response to systemic therapy [1, 3, 23, 29–31]. From a clinical perspective, there is an unmet need for effective CMS4-targeting therapies. The vast majority of GISTs have activating mutations in KIT, which drive tumorigenesis. Indeed, the KIT inhibitor imatinib has revolutionized the treatment of GISTs [32]. Our work suggests that high expression of (non-mutated) KIT promotes aggressive behavior in CRC models and, therefore, that imatinib may have value as a CMS4-targeting drug. The effect of Imatinib treatment on CMS4 CRC was assessed in the clinical proof-of-concept study ImPACCT, in which patients with newly-diagnosed CMS4 CRC were treated with imatinib two weeks prior surgery [6, 33]. By analysis of pre- and post-treatment samples, we recently demonstrated that imatinib mitigates the aggressive features of CMS4 CRC in this clinical study (Peters et al., submitted [34]), lending support for the concept of KIT-targeted treatment in the clinical management of CMS4 CRC [35].

One of the most characteristic features of CMS4 CRC is a high level of TGFβ-signaling in the tumor stroma [1]. A stromal TGFβ program predicts CRC relapse [36] and high expression of TGFβ in tissues and serum is associated with worse overall survival and recurrence in CRC patients undergoing surgery [37, 38]. In a spontaneous metastasis mouse model, pharmacological blockade of TGFβ stromal signaling prevented metastasis initiation [36]. These data indicate that TGFβ in the tumor microenvironment induces a pro-metastatic program. Our data show that *KIT* expression causes a phenotypic shift from CMS2 to CMS4 generating tumors with increased stromal content and high expression of TGFβ.

Mutational inactivation of the TGFβ signaling pathway is observed in ~50% of all CRCs, causing tumor cells to escape the tumor-suppressive effects of this pathway [39]. Indeed, PDOs with a mutated TGFB-pathway grow independently of the TGFβRII inhibitor A83-01, while suppression of TGFβRII activity is essential to maintain the regenerative capacity of PDOs with a wild-type TGFβ-pathway [40]. Similarly, the addition of A83-01 stimulates the formation of mouse normal colon organoids [41]. In addition, genetic inactivation of the TGFβ receptor 2 in intestinal epithelial cells is sufficient to cause the formation of invasive tumors in the context of chronic inflammation [42]. These studies highlight that suppressing TGFB signaling in epithelial intestinal cancer cells promotes tumor initiation and progression. Thus, tumors with a wild-type TGFβ pathway must activate tumor cell-intrinsic mechanisms that allow them to evade tumor-suppressive TGFb signaling [39].

Several mechanisms may contribute to overcoming TGFβ-induced tumor suppression. For example, mutations in *KRAS* increases resistance against TGFβ-induced cell-death by inhibiting the pro-apoptotic protein Bim [43]. In addition, *RAC1B* (GTPase *RAC1* splice isoform) confers protection against TGFβ-induced apoptosis by suppressing transcriptional output of the pathway, including the pro-apoptotic TGFβ effector gene BIM [44].

In conclusion, our study identifies high KIT expression as a third potential mechanism that is utilized by tumor cells to evade growth suppression by TGFβ, presumably by downregulating one of the core signaling components in the pathway, SMAD2. Our findings support a model in which KIT stimulates TGFβ expression to promote stroma formation and activation—resulting in CRC progression [36]—while simultaneously protecting the tumor cells against TGFβ-induced growth inhibition through suppression of SMAD2. Pharmacological inhibition of KIT may therefore represent an attractive approach to target the subgroup of KIT-expressing stroma-rich CRC [11, 12, 17].

## Supplementary information

Revised_Supplementary_Clean

Supplementary Figure 1

Supplementary Figure 2

Supplementary Figure 3

Supplementary Figure 4

Supplementary Figure 5

Supplementary Figure 6

Supplementary Figure 7

Supplementary Table 1

Supplementary Table 2

Supplementary Table 3

Supplementary Table 4

Supplementary Table 5

CDD Checklist

WB_Fig2D_1

WB_Fig2D_2

WB_Fig2H_1

WB_Fig2H_2

WB_Fig6G

WB_Fig6G_2

WB_Fig6G_3

## Data Availability

All data generated or analyzed during this study are available from the corresponding author on reasonable request.

## Code availability

Used code scripts are available from the corresponding author on reasonable request.
