# Supplementary material for: KIT promotes tumor stroma formation and counteracts tumor-suppressive TGFβ signaling in colorectal cancer
Source: Cell Death Dis. 2022 Jul 16;13(7):617. doi: 10.1038/s41419-022-05078-z (PMC9288482; doi:10.1038/s41419-022-05078-z)
Supplement: Supplementary file 1 — Revised_Supplementary_Clean [file 41419_2022_5078_MOESM1_ESM.docx]

Supplementary

**KIT promotes tumor stroma formation and counteracts tumor-suppressive TGFß signaling in colorectal cancer**

Emre Küçükköse, Niek A. Peters, Inge Ubink, Veere A.M. van Keulen, Roxanna Daghighian, André Verheem, Jamila Laoukili and Onno Kranenburg

**Materials and methods**

**Immunofluorescence**

PDOs were cultured for 5 days and harvested using 2 mg/mL Dispase type II (Sigma-Aldrich, Zijndrecht, The Netherlands, #D4693) by 15 min incubation at 37°C. After washout of Dispase type II with PBS (Sigma-Aldrich, Zwijndrecht, The Netherlands), the organoids were fixed in 4% formaldehyde solution at room temperature (RT) for 20 min. The formaldehyde solution was aspirated and the PDOs were incubated 30 min at RT in 500 µl PBS-Triton 0.5% solution. Subsequently, the solution was aspirated after centrifugation (500G, 3 min) and PDOs were incubated in 500 µl blocking solution PBS-Tween 0.1% with 5% BSA. The PDOs were incubated with primary antibodies (**Supplementary table S3**) overnight at 4°C. The next day, the PDOs were washed with PBS-Tween 0.1% solution in total two times and incubated with secondary antibodies (**Supplementary table S3**) for 2 hours at RT in a dark environment. The PDOs were washed once in PBS-Tween 0.1% and PBS solutions and Cytocentrifuged (500G for 5 min) on a glass slide and mounted with 5 µl ProLong™ Gold Antifade Mountant (P36930, Thermo Fisher Scientific, Breda, The Netherlands).

**Western blot**

The PDOs were harvested as described above and lysed using Laemmli buffer (10% glycerol, 2% SDS, 63 mM Tris-HCl pH6.8). A total protein concentration was determined using the Lowry method (1). Proteins were separated via SDS-PAGE gel-electrophoresis and transferred on 0.2 µm Nitrocellulose membranes using a Trans-blot Turbo transfer system (1704158, BioRad, Lunteren, The Netherlands). PageRuler™ Plus Prestained Protein Ladder (26620, Thermo Fisher Scientific, Breda, The Netherlands) was used. After 1 h blocking of unspecific binding with 5% BSA in 1× TBS-0.1% Tween solution, membranes were incubated with primary antibodies overnight at 4°C (**Supplementary table S3**). Next, membranes were incubated with HRP-conjugated secondary antibodies for 1 h at RT. After each antibody incubation, membranes were washed three times with 1× TBS for 5 min at RT. Protein detection was performed using enhanced chemiluminescence (ECL) reagents (Amersham ECL, GE Healthcare, RPN2235/2209, Chicago, IL, USA).

**Transcriptome profiling using RNA sequencing**

For transcriptome profiling of PDOs or subcutaneous tumors, total RNA was isolated using Qiagen RNeasy kit (Qiagen, 74104, Venlo, The Netherlands) according to the manufacturer’s instructions. For each PDO, three RNA replicates were used for sequencing, isolated from three different organoid culture passages. The PDOs were harvested as described above and lysed using RLT RNA lysis buffer. RNA quality was assessed using an Agilent 2100 Bioanalyzer. RNA samples with high integrity numbers (RIN 9–10) were used for sequencing. Sequencing was performed by the Utrecht Sequencing Facility using the Nextseq500 platform, high output 1 × 75 bp run type. RNA expression data were analyzed using the R2: Genomics Analysis and Visualization Platform (<http://r2.amc.nl>).

***In vivo* subcutaneous tumorigenesis**

Animal experiments were approved by the Competent Authority, The Netherlands (License number AVD115002016614), which is advised by the Animal Ethics Committee. Animal work protocols were approved by the Animal Welfare Body and were performed in accordance with the Dutch Law on Animal Experiments and the European Directive 2010/63/EU. Healthy 8–10 week old 25–30 g male *NOD.Cg-Prkdc^scid^ Il2rg^tm1Wjl^/SzJ* (NSG) mice mice were supplied by Charles River. Animals were randomly allocated in groups of three mice into individually ventilated cages. Animals were kept at RT under 12 h light/dark cycles and received standard chow pellets and water ad libitum. Five days old PDOs were dissociated into single-cells by TrypLE Express incubation for 5 min at 37°C. A cell-matrigel (1:1) suspension was prepared with 5 × 10^6^ cells/ml. Using a 1 ml syringe and 25G 0.5 × 16 mm needles, 100 µl of the suspension were subcutaneously injected into the right flank of animals. Animal welfare was monitored by physical appearance, behavior, and body weight. Animals were sacrificed when the tumor volume (V) reached 1.5 mm^3^, as measured by V = 1/2 × (smaller diameter^2^ × larger diameter). Tumor tissue was harvested for further immunohistochemical analyzes.

Sample sizes are outlined in the figure legends. Mice were randomly assigned to experimental groups. No blinding method was used for injection. No animals were excluded from downstream analysis.

**Immunohistochemistry (IHC)**

Subcutaneous tumor tissue was fixed in 4% formaldehyde solution and paraffin embedded. Sections of 4 µm thickness were made. Prior to the staining, tissue sections were deparaffinized and rehydrated. Endogenous peroxidase activity was blocked with 1.5% hydrogen peroxide for 30 min. Heat-induced antigen retrieval was carried out using citrate buffer pH6.0 for 20 min, followed by the cooling of tissue sections for 20 min. Sections were incubated overnight at 4°C with primary antibodies (**Supplementary table 3**) diluted in PBS with 0.1% Sodium azide and 3% BSA. The next day, sections were washed three times with 0.05% Tween-PBS solution for 5 min, followed by a 1 h incubation with the HRP-conjugated secondary antibody. Subsequently, sections were washed three times with 1× PBS for 5 min and developed with 3,3’-Diaminobenzidine (DAB) chromogen for 10 min at RT in the dark. Sections were rinsed under running tap water for 10 min and counterstained with hematoxylin, followed by dehydration and mounting.

**Orthotopic implantation in the caecum of NSG mice**

In order to evaluate the tumorigenic capacity of the PDOs, we made use of the murine orthotopic caecum-implantation model (2). In summary, day before implantation, PDOs were dissociated into single cells and 2.5 × 10^5^ cells were plated in 10 μL drops of neutralized Rat Tail High Concentrated Type I Collagen (Corning, C3867). PDOs were allowed to recover overnight at 37°C, 5% (vol/vol) CO2. *NOD.Cg-Prkdc^scid^ Il2rg^tm1Wjl^/SzJ* (NSG) mice were treated with a s.c. dose of Carprofen (5 mg/kg, RimadylTM) 30 min before surgery and were subsequently sedated by using isoflurane inhalation anesthesia [∼2% (vol/vol) isoflurane/O2 mixture]. The caecum was exteriorized through a midline abdominal incision and a single collagen drop containing organoids was surgically transplanted in the cecal submucosa. Carprofen was administrated s.c. post 24 h surgery. Animal welfare was monitored by physical appearance, behavior, and body weight.

Sample sizes were n = 7 or 8 mice for PDO2^CONTROL^ and PDO2^KIT^, respectively. Power analyses were carried out prior to experiments being carried out to determine the minimum number of animals required for each experiment. These analyses were informed by previous and / or preliminary experiments. Animals were house vested in groups of four. Every group (i.e. cage) contained mixture of mice transplanted with both PDOs. No animals were excluded from downstream analysis. The investigator was blinded to the group allocation of the animals during the experiment.

**Supplementary legends**

**Supplementary Fig. 1 – Differential exon expression analysis.** RNA-seq data of PDO1 (red) and PDO1^KIT-KO^ (blue) were analyzed to determine the exon usage of *KIT* using the DEXSeq package (3). Left panel demonstrates the expression of every feature in KIT gene (ENSG00000157404). Right dot plot demonstrate the exon usage (%) of PDO1^KIT-KO^ relative to the exon usage in PDO1^CONTROL^. Vertical red lines represent the mean.

**Supplementary Fig. 2 – Immunohistochemistry evaluation of TGFb1 in subcutaneous tumors.** Histological images of TGFb1 in s.c. tumors and corresponding quantification.

**Supplementary Fig. 3 – Mesenchymal-like tumors have reduced oxidative phosphorylation.** Scatter plot showing inverse correlation between hallmark signatures “oxidative phosphorylation” and “epithelial-mesenchymal-transition” for subcutaneous tumors and CRC tumors in the CMS-3232 cohort (4).

**Supplementary Fig. 4 – KIT-dependency signature correlates with the KIT-co-expression signature.** Scatter plot showing a correlation between PDO-initiated KIT-dependency signature and “KIT-co-expressed” signature (5) for CRC tumors in the CMS-3232 cohort (4).

**Supplementary Fig. 5 – Reduced regenerative capacity in KIT-negative models upon A83-01 omission.** Brightfield images of PDO1^CONTROL^, PDO1^KIT-KO^, PDO2^CONTROL^, and PDO2^KIT^ normal CRC culture medium and A83-01 depleted medium. Scale bar is 500 µm.

**Supplementary Fig. 6 – Dasatinib or Imatinib renders PDO1 sensitive to TGFβ-mediated growth inhibition.** Quantification of a regenerative capacity assay in which 1,000 single cells are seeded in control CRC culture medium or A83-01 depleted medium (-TGFBRI), with or without Dasatinib (Selleckchem, S1021, 100 nM), Imatinib (Santa Cruz Biotechnology, sc-202180A, 5 µM) and/or stem-cell factor (SCF, Peptrotech, 300-07, 100 ng/µl). The number of organoids are counted after two weeks. N = two different experiments with at least three technical replicates in each.

**Supplementary Fig. 7 – Galunisertib increased the regenerative capacity in KIT-negative and TGFB-pathway wild-type models.** Quantification of a regenerative capacity assay in which 1,000 single cells are seeded in A83-01 depleted medium (-TGFBRI), A83-01 or Galunisertib (LY2157299, Selleckchem, S2230, 500 nM) presence. The number of organoids are counted after two weeks. N = two different experiments with at least three technical replicates in each.

**Supplementary Table 1 – Culture medium composition for colorectal cancer organoids**

**Supplementary Table 2 – Oligo's used in this study**

**Supplementary Table 3 – Antibodies used in this study**

**Supplementary Table 4 – Differential gene expression analyses between KIT-negative and KIT-positive subcutaneous tumors**

**Supplementary Table 5 – Differential gene expression analyses between TGFB-low and TGFB-high CRC tumors from CMS3232 dataset**

**Supplementary References**

1. Lowry OH, Rosebrough NJ, Farr AL, Randall RJ. Protein measurement with the Folin phenol reagent. J Biol Chem. 1951;193(1):265-75.

2. Fumagalli A, Suijkerbuijk SJE, Begthel H, Beerling E, Oost KC, Snippert HJ, et al. A surgical orthotopic organoid transplantation approach in mice to visualize and study colorectal cancer progression. Nat Protoc. 2018;13(2):235-47.

3. Anders S, Reyes A, Huber W. Detecting differential usage of exons from RNA-seq data. Genome Res. 2012;22(10):2008-17.

4. Guinney J, Dienstmann R, Wang X, de Reynies A, Schlicker A, Soneson C, et al. The consensus molecular subtypes of colorectal cancer. Nat Med. 2015;21(11):1350-6.

5. Fatrai S, van Schelven SJ, Ubink I, Govaert KM, Raats D, Koster J, et al. Maintenance of Clonogenic KIT(+) Human Colon Tumor Cells Requires Secretion of Stem Cell Factor by Differentiated Tumor Cells. Gastroenterology. 2015;149(3):692-704.
