# Supplementary figures and images for: KIT promotes tumor stroma formation and counteracts tumor-suppressive TGFβ signaling in colorectal cancer
Source: Cell Death Dis. 2022 Jul 16;13(7):617. doi: 10.1038/s41419-022-05078-z (PMC9288482; doi:10.1038/s41419-022-05078-z)

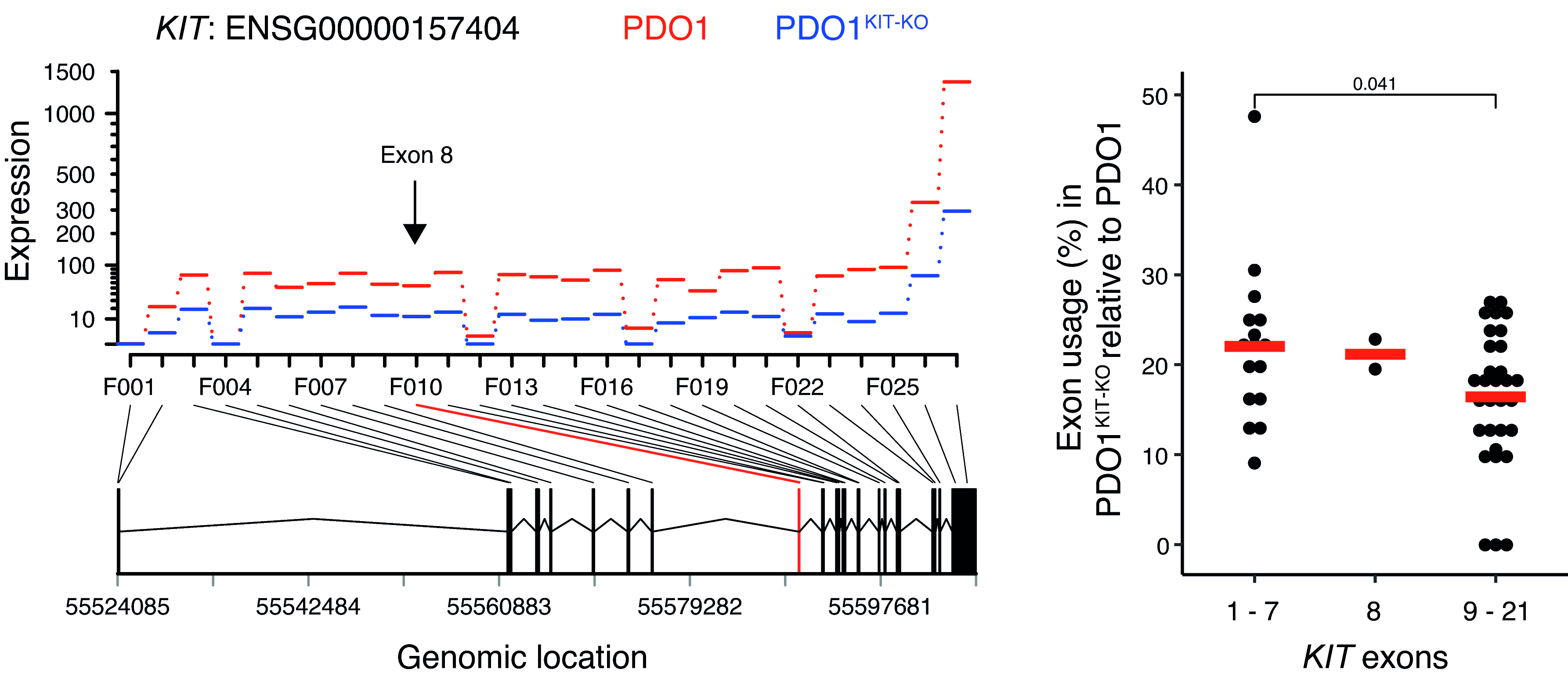

Supplement: Supplementary file 2 — Supplementary Figure 1 [file 41419_2022_5078_MOESM2_ESM.tif]

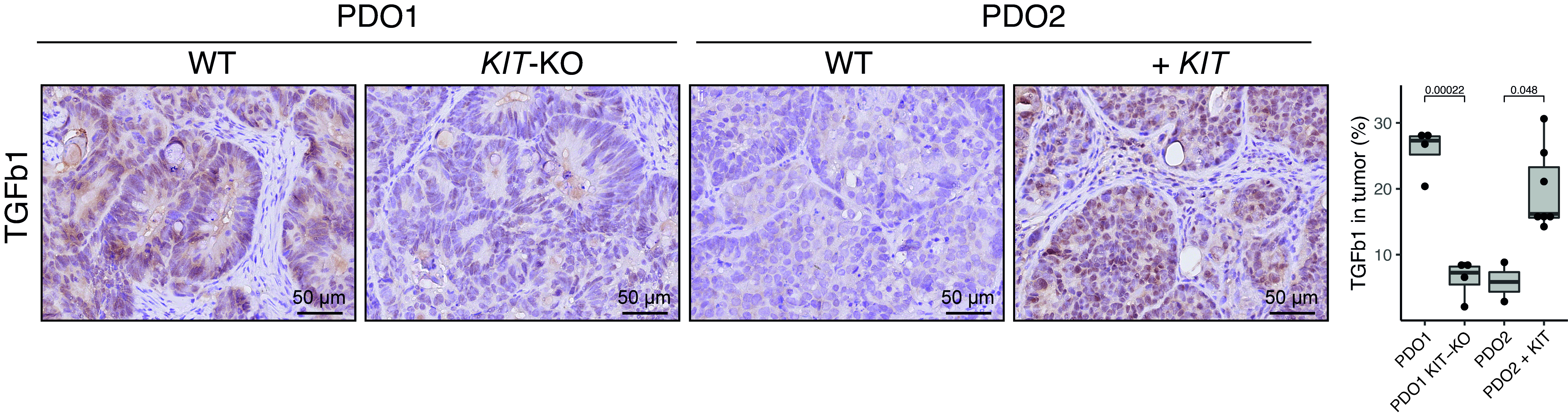

Supplement: Supplementary file 3 — Supplementary Figure 2 [file 41419_2022_5078_MOESM3_ESM.tif]

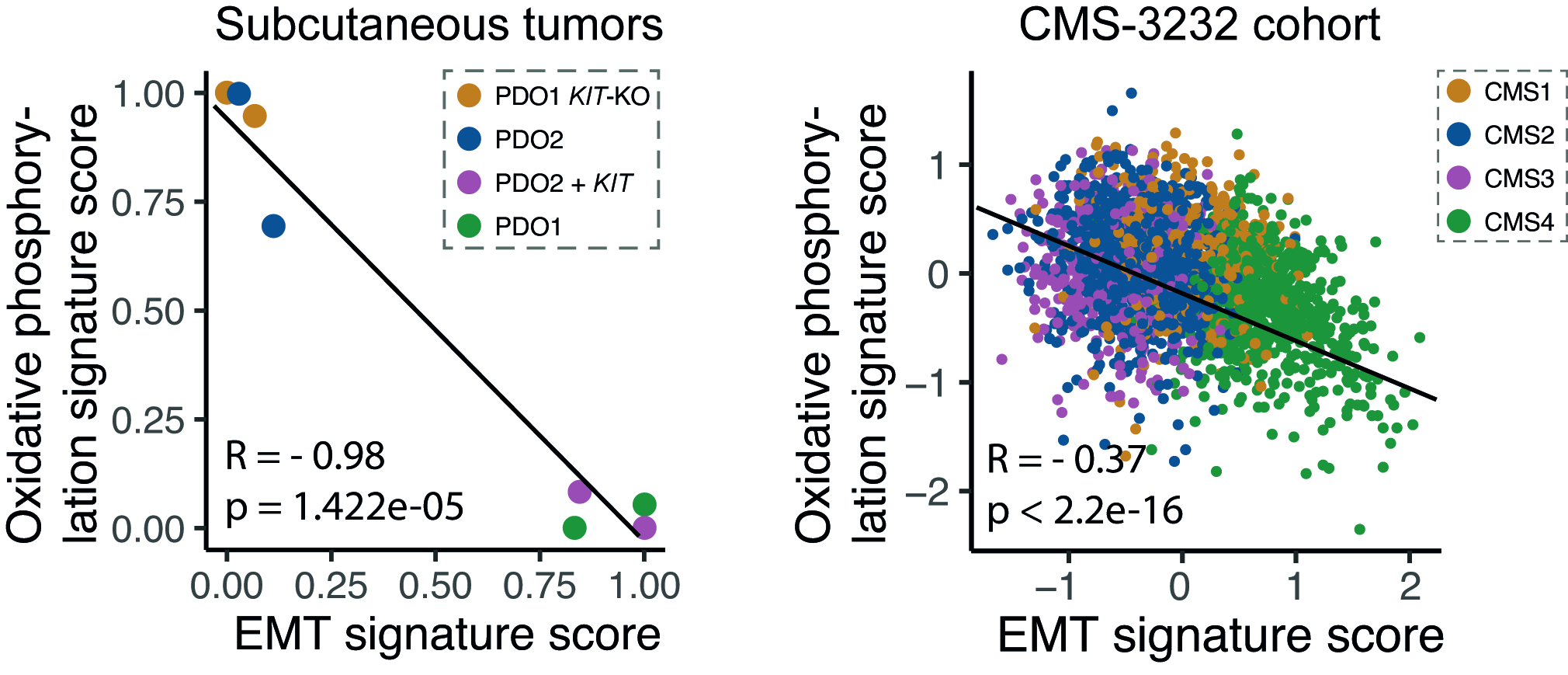

Supplement: Supplementary file 4 — Supplementary Figure 3 [file 41419_2022_5078_MOESM4_ESM.tif]

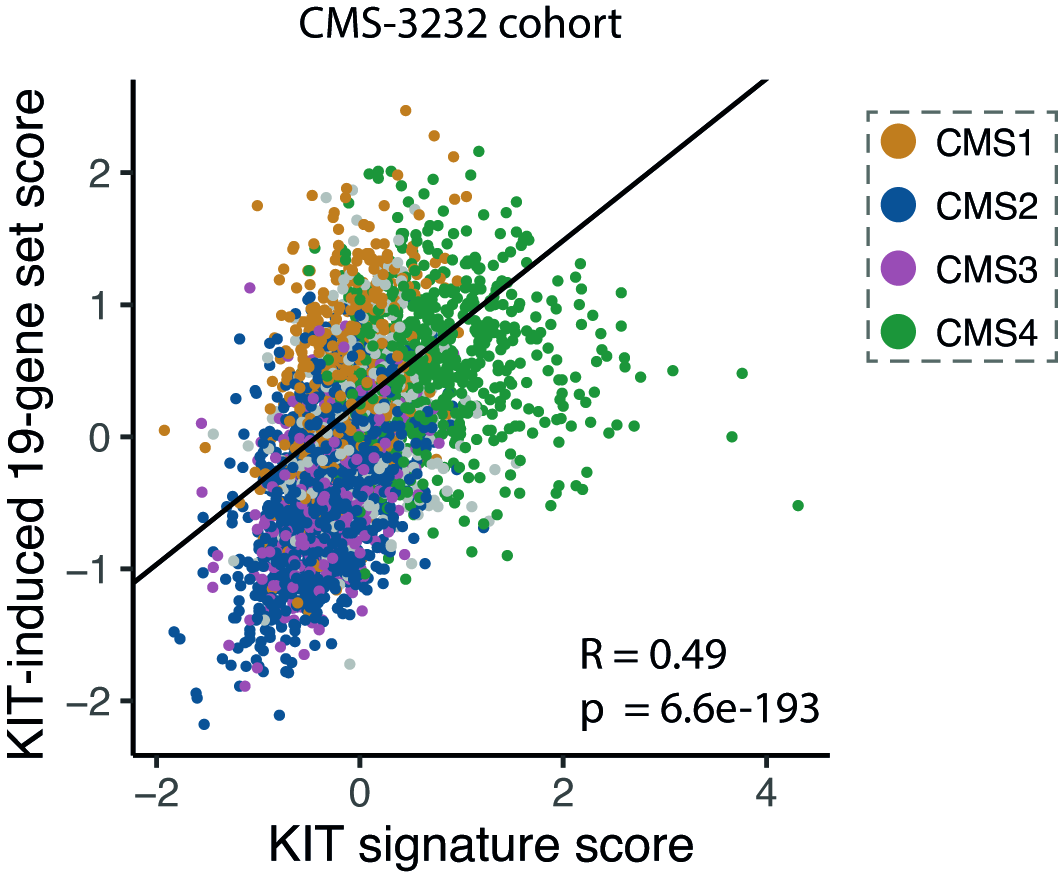

Supplement: Supplementary file 5 — Supplementary Figure 4 [file 41419_2022_5078_MOESM5_ESM.tif]

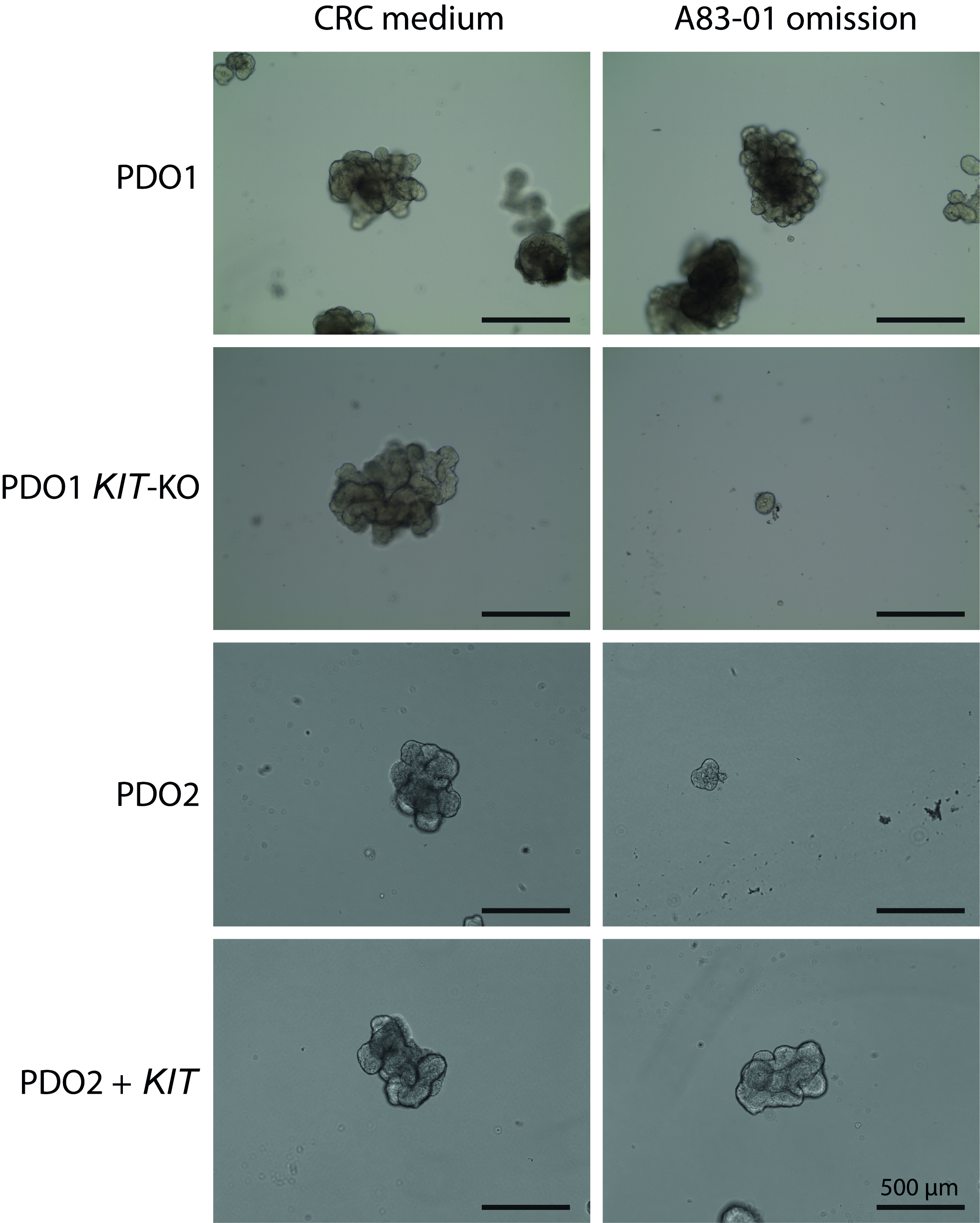

Supplement: Supplementary file 6 — Supplementary Figure 5 [file 41419_2022_5078_MOESM6_ESM.tif]

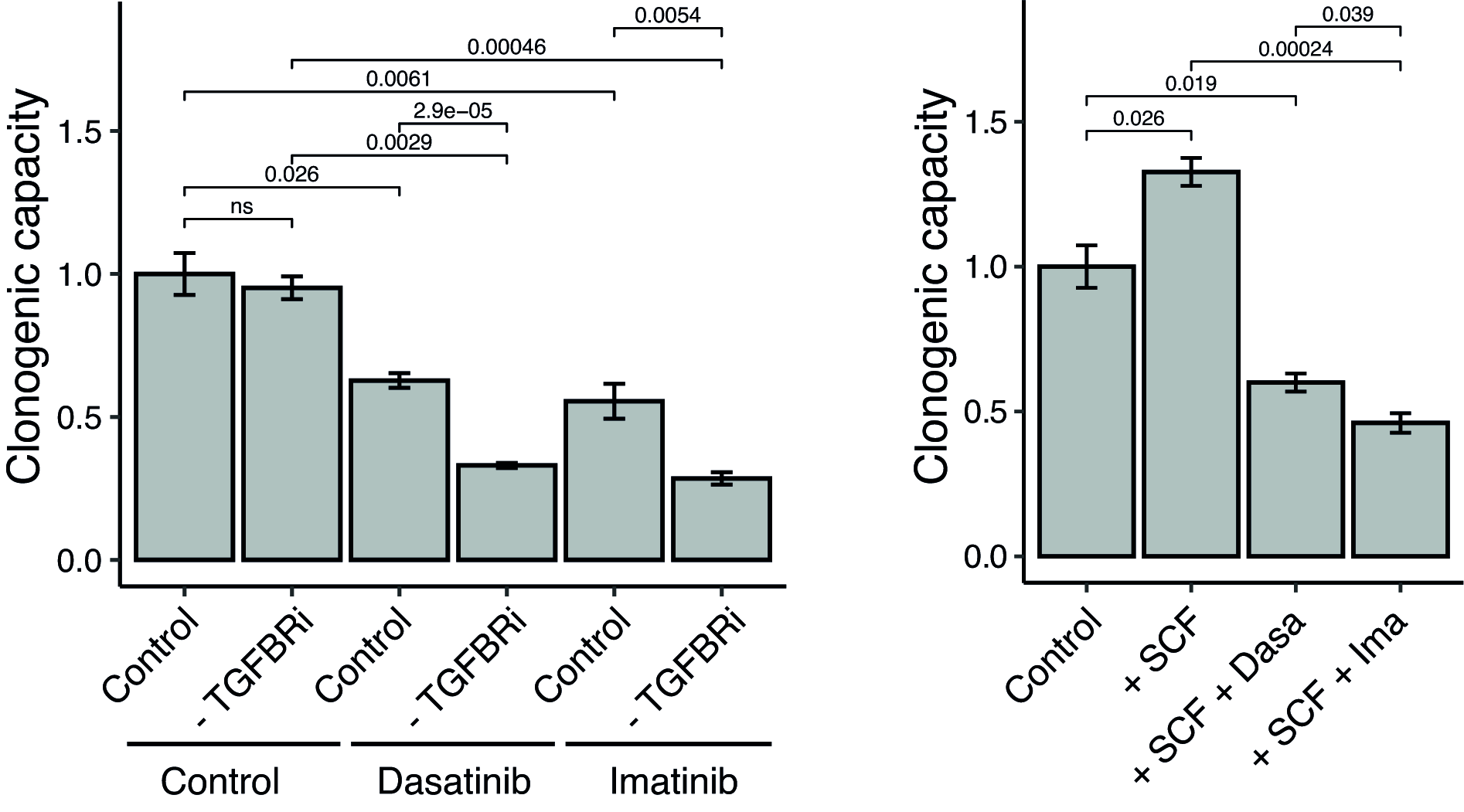

Supplement: Supplementary file 7 — Supplementary Figure 6 [file 41419_2022_5078_MOESM7_ESM.tif]

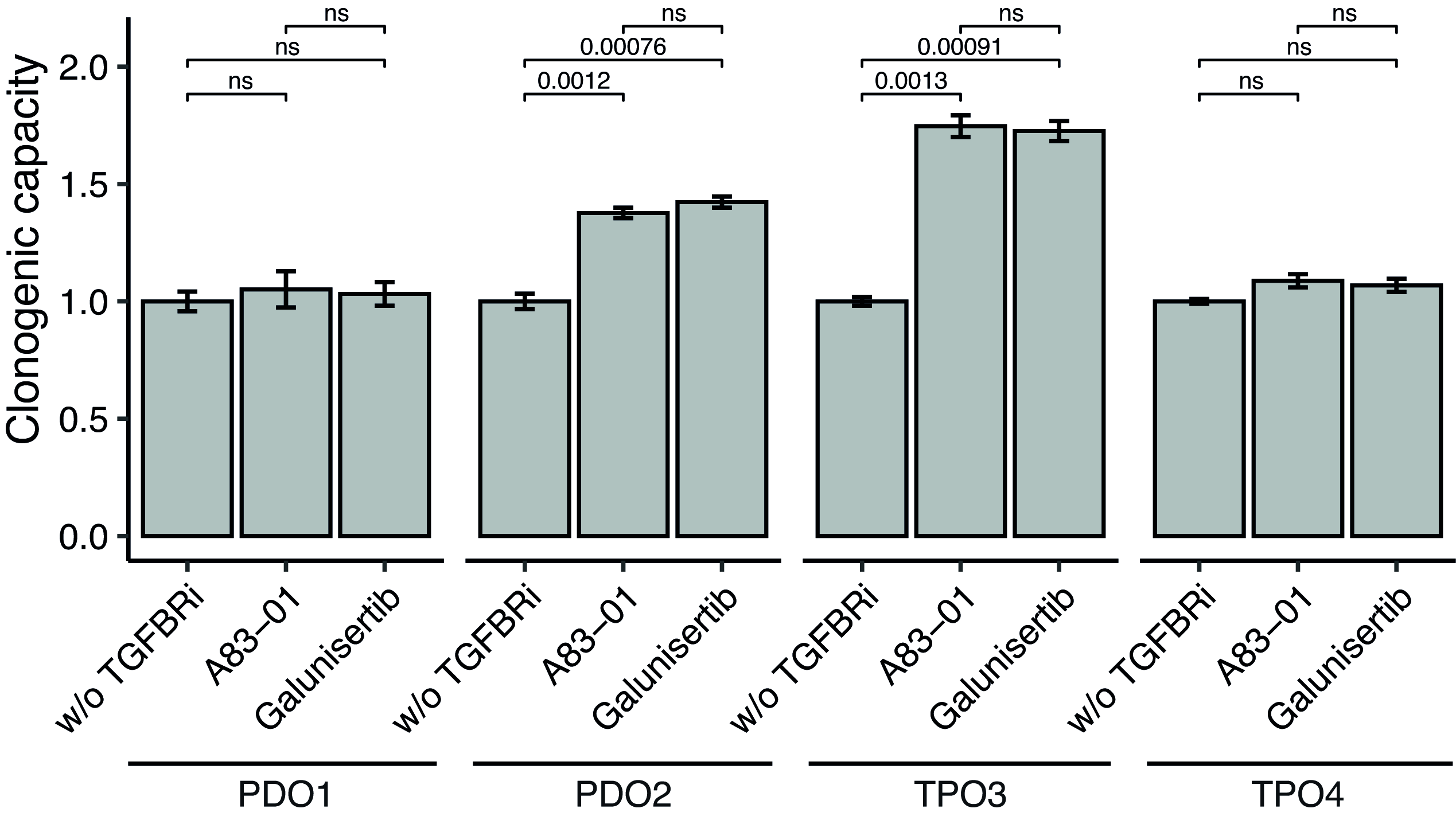

Supplement: Supplementary file 8 — Supplementary Figure 7 [file 41419_2022_5078_MOESM8_ESM.tif]

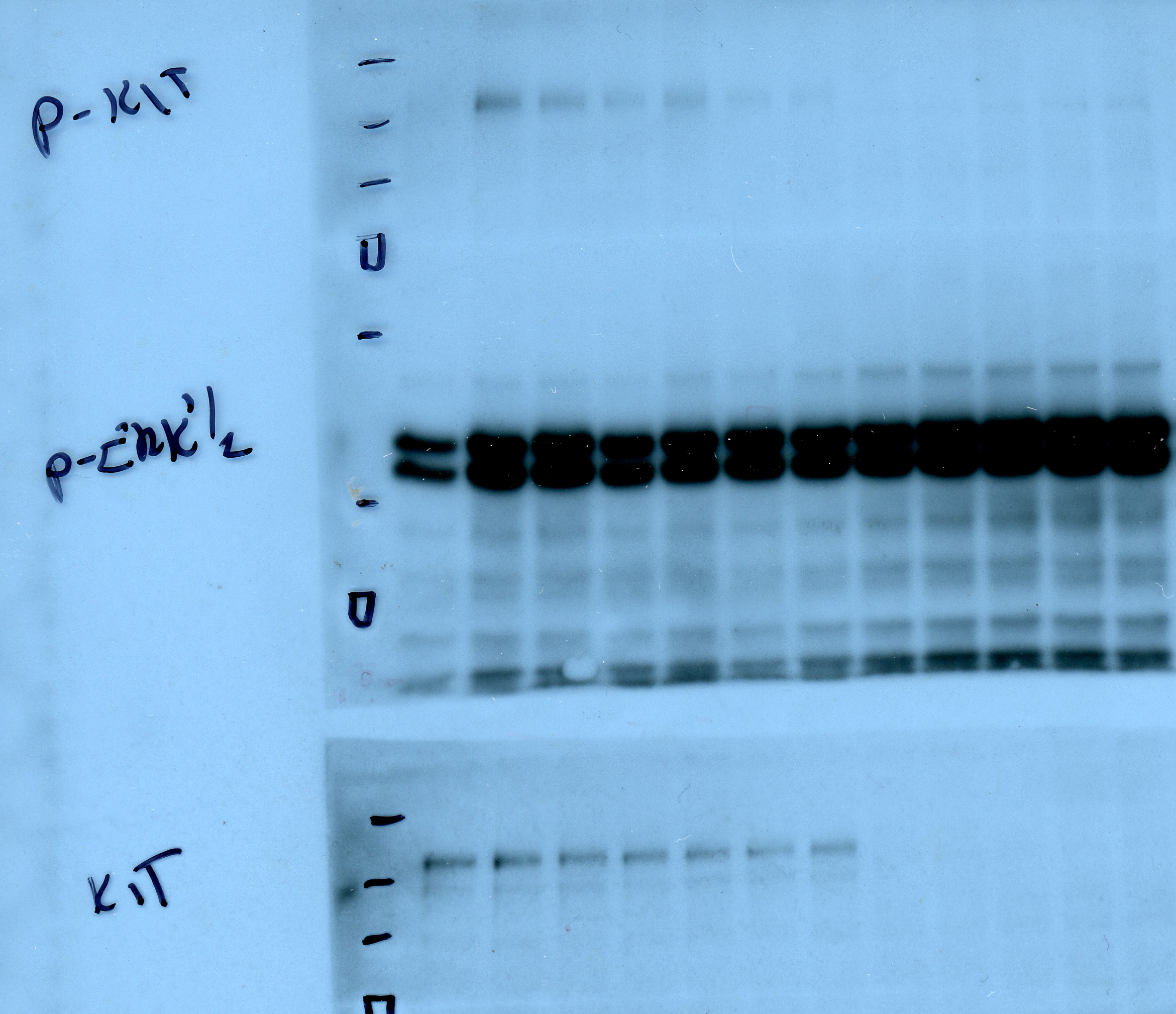

Supplement: Supplementary file 15 — WB_Fig2D_1 [file 41419_2022_5078_MOESM15_ESM.png]

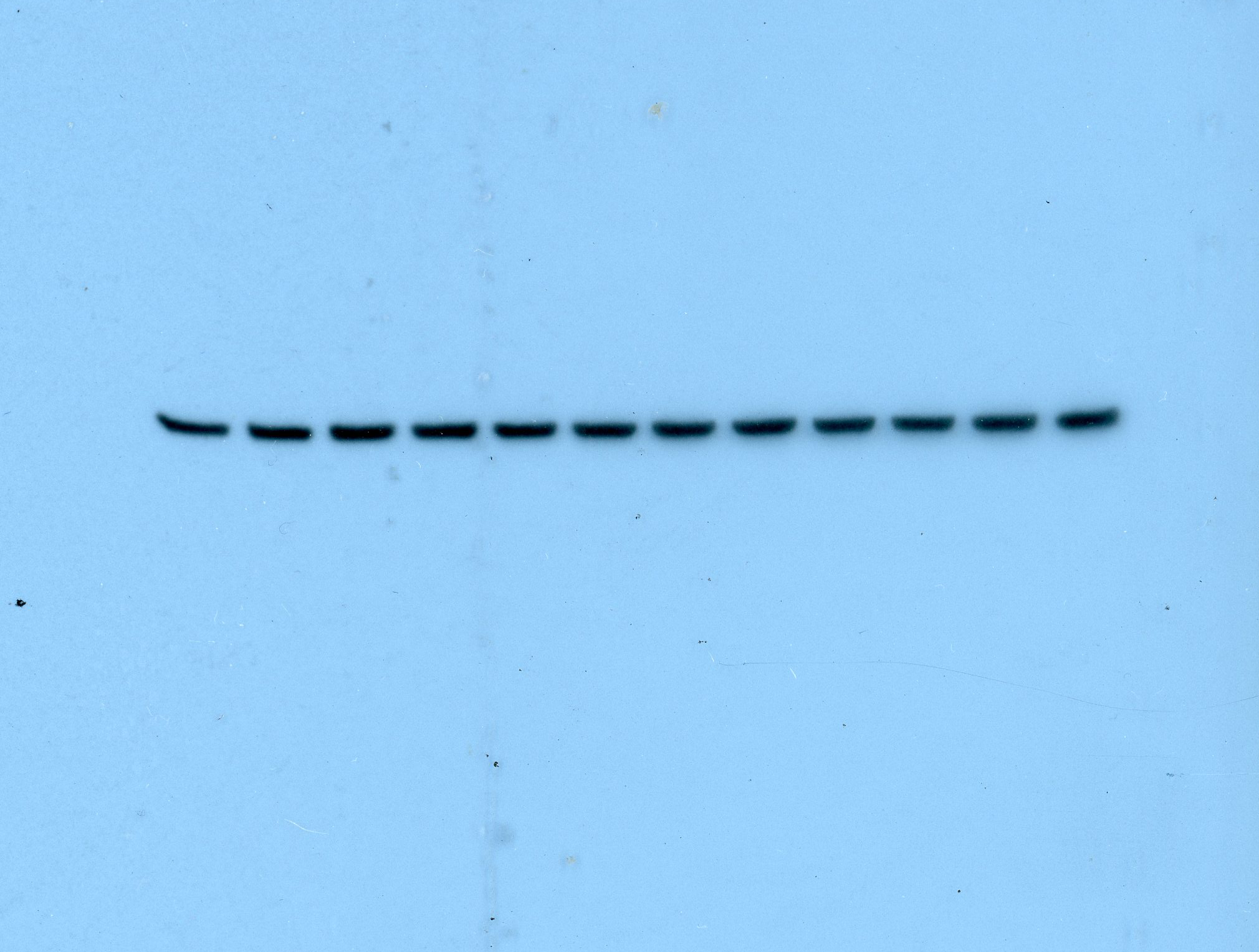

Supplement: Supplementary file 16 — WB_Fig2D_2 [file 41419_2022_5078_MOESM16_ESM.png]

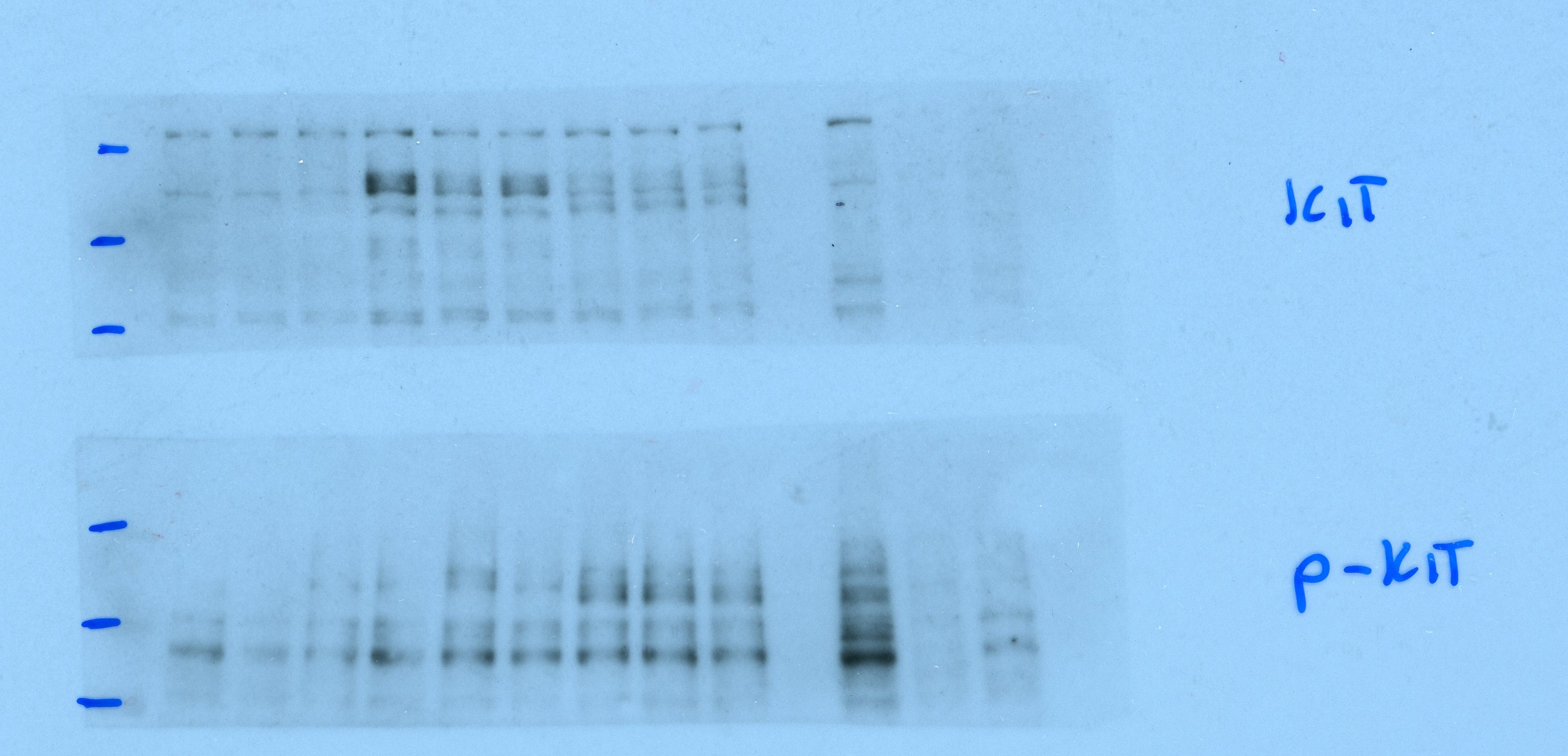

Supplement: Supplementary file 17 — WB_Fig2H_1 [file 41419_2022_5078_MOESM17_ESM.png]

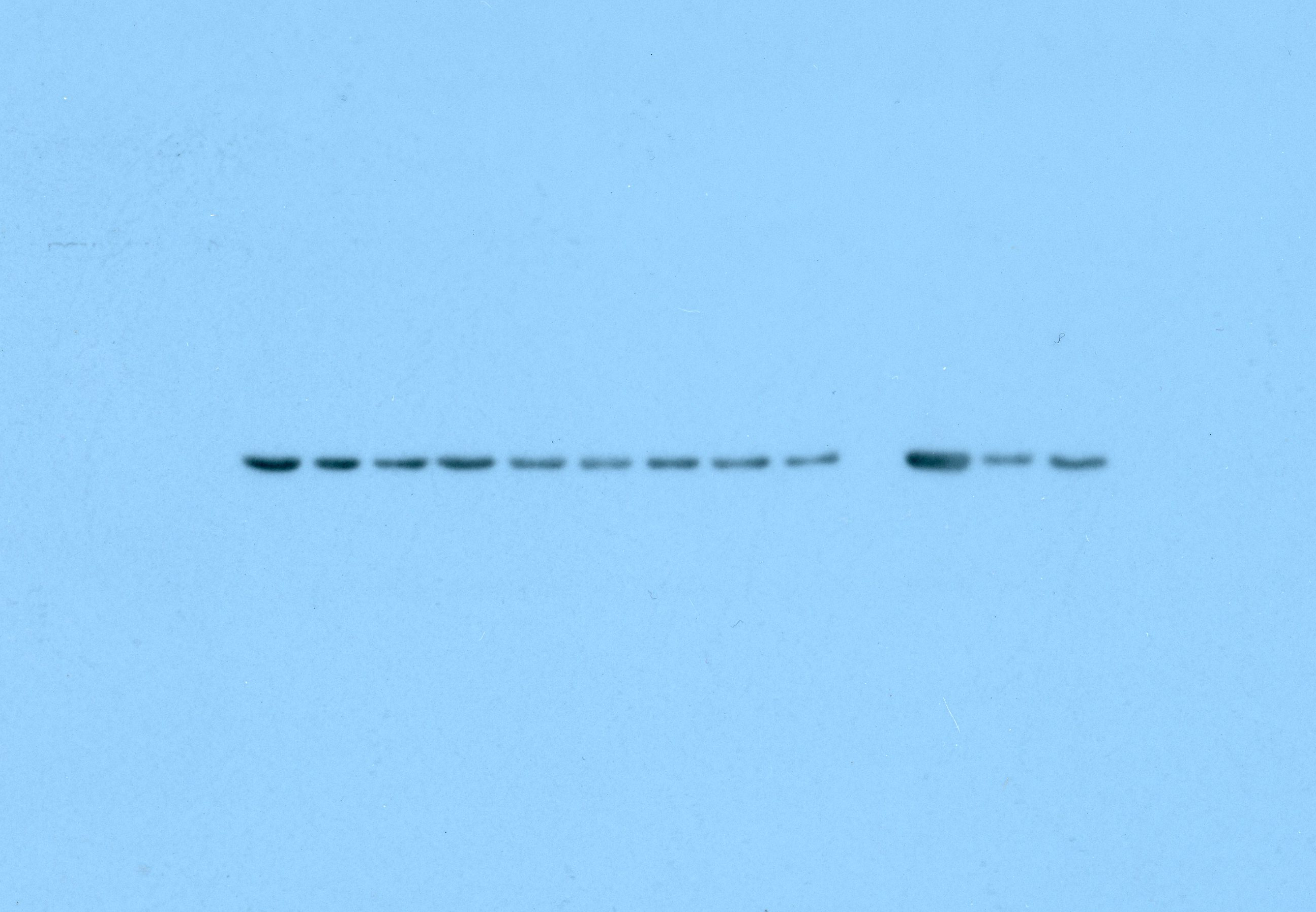

Supplement: Supplementary file 18 — WB_Fig2H_2 [file 41419_2022_5078_MOESM18_ESM.png]

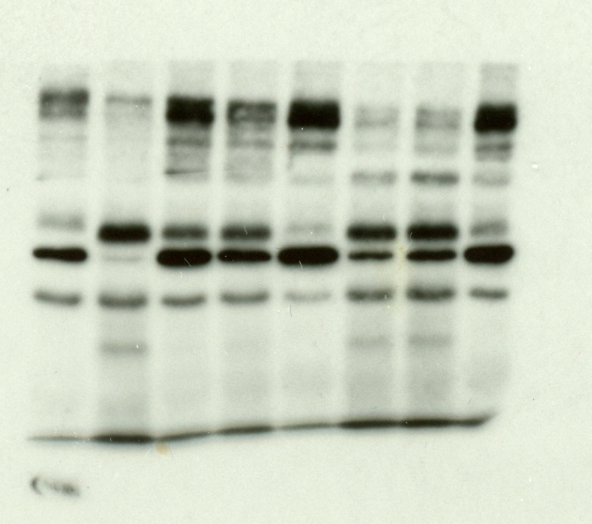

Supplement: Supplementary file 19 — WB_Fig6G [file 41419_2022_5078_MOESM19_ESM.png]

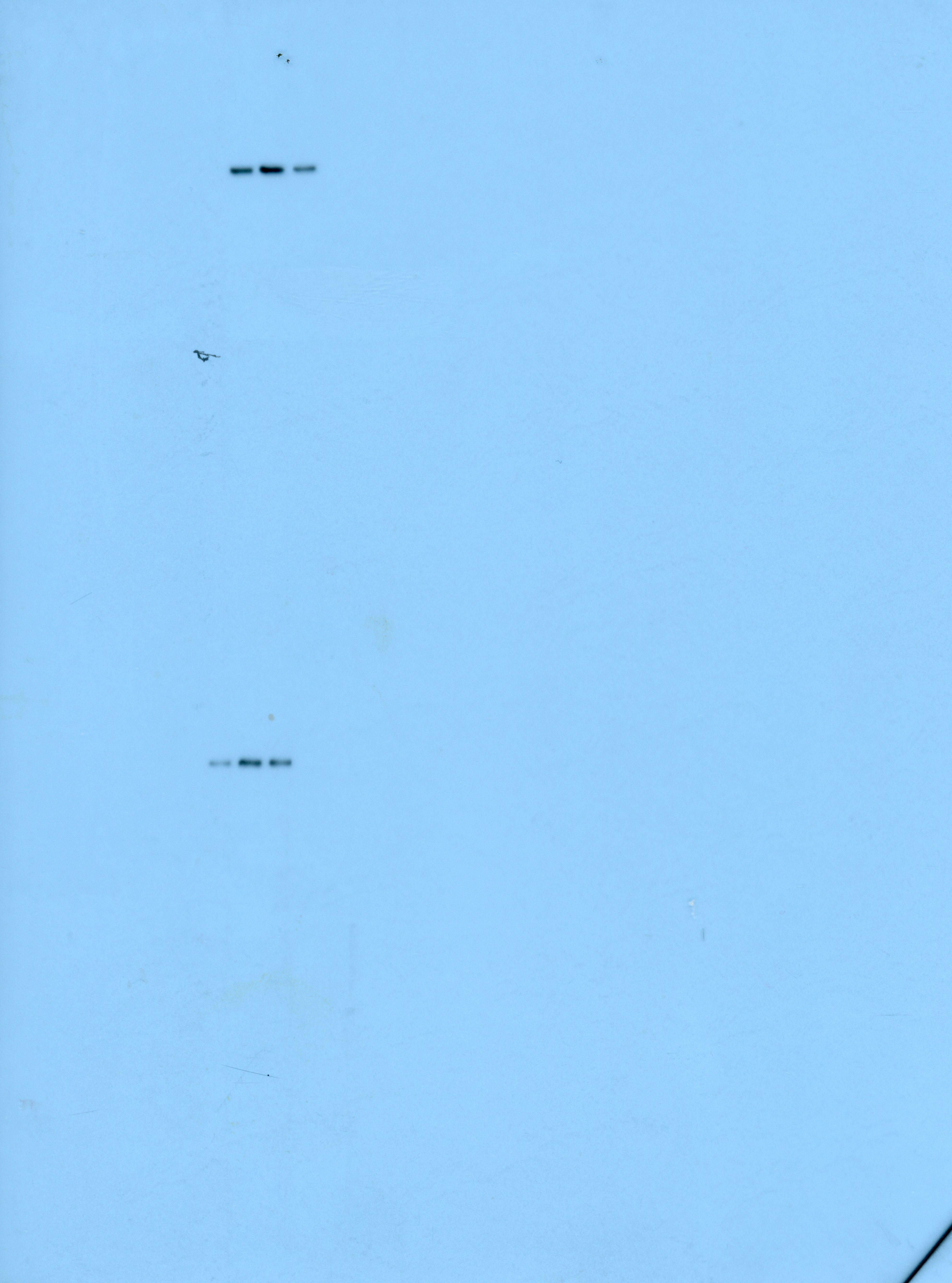

Supplement: Supplementary file 20 — WB_Fig6G_2 [file 41419_2022_5078_MOESM20_ESM.tif]

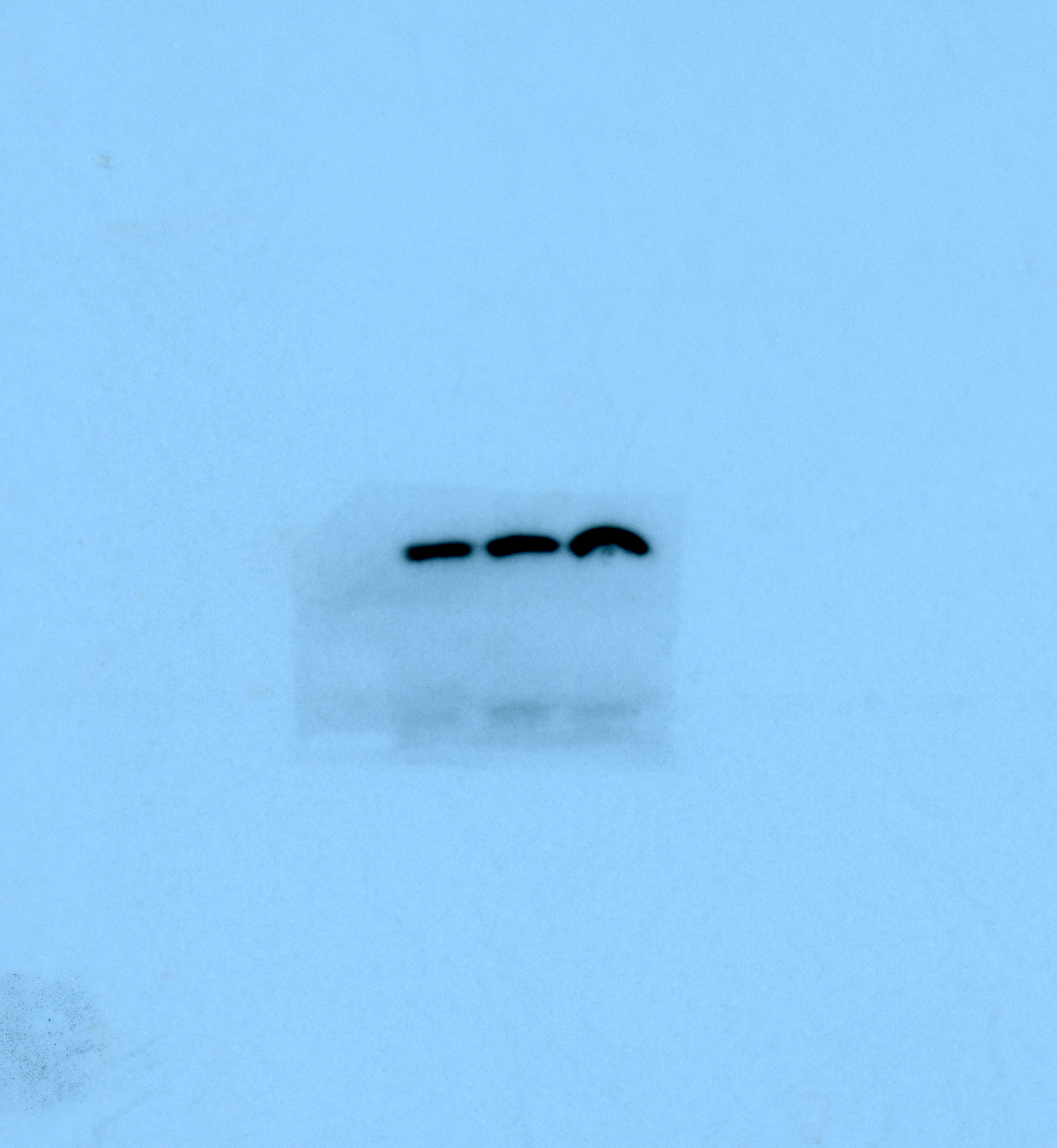

Supplement: Supplementary file 21 — WB_Fig6G_3 [file 41419_2022_5078_MOESM21_ESM.tif]
